# Prevalence and genotypic identification of *Cryptosporidium* spp., *Giardia duodenalis* and *Enterocytozoon bieneusi* in pre-weaned dairy calves in Guangdong, China

**DOI:** 10.1186/s13071-019-3310-5

**Published:** 2019-01-17

**Authors:** Yuanyuan Feng, Xiaoqing Gong, Kexin Zhu, Na Li, Zhengjie Yu, Yaqiong Guo, Yabiao Weng, Martin Kváč, Yaoyu Feng, Lihua Xiao

**Affiliations:** 1 State Key Laboratory of Bioreactor Engineering, School of Resource and Environmental, East China University of Science and Technology, Shanghai, 200237 China; 2 Key Laboratory of Zoonosis of Ministry of Agriculture, College of Veterinary Medicine, South China Agricultural University, Guangzhou, 510642 China; 3 Institute of Parasitology, Biology Centre of the Academy of Sciences of the Czech Republic, České Budějovice, Czech Republic

**Keywords:** *Cryptosporidium*, *Giardia duodenalis*, *Enterocytozoon bieneusi*, Molecular epidemiology

## Abstract

**Background:**

*Cryptosporidium* spp., *Giardia duodenalis* and *Enterocytozoon bieneusi* are common enteric pathogens in humans and animals. Data on the transmission of these pathogens are scarce from Guangdong, China, which has a subtropical monsoon climate and is the epicenter for many emerging infectious diseases. This study was conducted to better understand the prevalence and identity of the three pathogens in pre-weaned dairy calves in Guangdong.

**Methods:**

The occurrence and genetic identity of three pathogens were analyzed by polymerase chain reaction. PCR-positive products were sequenced to determine the species and genotypes. A Chi-square test was used to compare the prevalence of pathogens among sampling dates, age groups, or clinical signs.

**Results:**

The detection rates of *Cryptosporidium* spp., *G. duodenalis* and *E. bieneusi* were 24.0% (93/388), 74.2% (288/388) and 15.7% (61/388), respectively. Three *Cryptosporidium* species were detected, including *C. bovis* (*n* = 73), *C. parvum* (*n* = 12) and *C. ryanae* (*n* = 7); one animal had concurrence of *C. bovis* and *C. parvum*. *C. parvum* was the dominant species during the first two weeks of life, whereas *C. bovis* and *C. ryanae* were mostly seen at 3–9 weeks of age. Sequence analysis identified the *C. parvum* as subtype IIdA19G1. Assemblage E (*n* = 282), assemblage A (*n* = 1), and concurrence of A and E (*n* = 5) were identified among *G. duodenalis*-positive animals using multilocus genotyping (MLG). Altogether, 15, 10 and 17 subtypes of assemblage E were observed at the *bg*, *gdh* and *tpi* loci, respectively, forming 49 assemblage E MLGs. The highest detection rate of *G. duodenalis* was found in winter. Sequence analysis identified genotypes J (*n* = 57), D (*n* = 3) and one concurrence of J and D among *E. bieneusi-*positive animals. The detection rate of *E. bieneusi* was significantly higher in spring (38.0%; 41/108) than in summer (7.2%; 8/111) and winter (7.1%; 12/169).

**Conclusions:**

These results indicate a common occurrence of *C. parvum* subtype IIdA19G1, *G. duodenalis* assemblage E, and *E. bieneusi* genotype J in pre-weaned dairy calves in Guangdong. More studies are needed to understand the unique genetic characteristics and zoonotic potential of the three enteric pathogens in the province.

## Background

*Cryptosporidium* spp., *Giardia duodenalis* and *Enterocytozoon bieneusi* are common zoonotic pathogens, causing diarrhea and other gastrointestinal illness in humans and animals and responsible for significant morbidity and mortality. In humans, they are transmitted by direct contact with infected persons (anthroponotic transmission) or animals (zoonotic transmission) or through consumption of contaminated food or water (food-borne or water-borne transmission) [1–4].

In case-control studies, contact with cattle has been implicated as a major risk factor in the epidemiology of human cryptosporidiosis [5]. A wide range of *Cryptosporidium* species have been identified in cattle, but studies worldwide have shown a common occurrence of *C. parvum*, *C. bovis*, *C. ryanae* and *C. andersoni* [3]. However, the prevalence of *C. parvum* in dairy cattle appears to be different between China and other countries [4, 6]. In China, pre-weaned dairy calves are mainly infected with *C. bovis* rather than *C. parvum* [7–13], although concurrence of *C. parvum* is increasingly detected [14–18]. Elsewhere in the world, *C. parvum* is responsible for over 90% of the infections in pre-weaned dairy calves [3]; only a few studies in Sweden, Egypt and Malaysia [19–21] showed *C. bovis* being relatively more common.

Subtyping of *C. parvum* targeting the 60-kD glycoprotein (*gp60*) gene identified mainly IIa and IId subtypes in dairy cattle [3]. Characterizations of *C. parvum* isolates in most industrialized countries of America, Europe and Asia suggest that pre-weaned dairy calves are almost exclusively infected with IIa subtypes, with IIaA15G2R1 as the most common subtype [3] while IId subtypes are only seen in Sweden, Egypt and Malaysia, where *C. bovis* is also commonly co-occurring in pre-weaned dairy calves [19–21]. In contrast, IId subtypes are common in pre-weaned calves in China, including IIdA14G1, IIdA15G1, IIdA17G1, IIdA19G1 and IIdA20G1 [10, 11, 13, 15, 16, 18]. Among them, IIdA15G1 and IIdA19G1 are the two major subtypes, responsible for over 90% of *C. parvum* infections [6].

Thus far, at least eight assemblages (A-H) of *G. duodenalis* have been identified. Among them, assemblages A and B have been found in humans and many other mammals whereas the remaining ones are mostly host-specific [2]. Most studies in Europe, America, Asia, Australia and Africa identified assemblage E as the genotype responsible for the majority of *G. duodenalis* infections in pre-weaned dairy cattle [12, 13, 17, 22–30], although in a few studies assemblages A and B were detected [31, 32]. Within assemblage A, there are two common sub-assemblages, AI and AII. The former is more commonly found in animals, whereas the latter is more commonly found in humans [2]. A few recent studies have also identified assemblage E in humans [33–35].

Over 250 *E. bieneusi* genotypes have been detected worldwide, including > 40 genotypes in cattle. They mostly belong to nine groups. Among the genotypes in cattle, at least 15, including eight genotypes in group 1 and seven genotypes in group 2, have been reported in humans [36–38], revealing the zoonotic potential of bovine *E. bieneusi* genotypes. The most prevalent genotypes in pre-weaned dairy calves worldwide are I, J and BEB4, all within group 2 [17, 27, 37, 39–42].

Pre-weaned dairy calves are the most common hosts of zoonotic *Cryptosporidium*, *G. duodenalis* and *E. bieneusi* genotypes. In China, these pathogens have been detected in pre-weaned dairy calves in many areas [8–13, 16–18, 24, 25, 27, 29, 32, 39–41, 43–46]. Guangdong is the epicenter of many emerging infectious diseases, and has intensive dairy farming and a subtropical monsoon climate with a warm and rainy spring, a long, hot and humid summer, and a mild and dry winter; the mean temperature in spring, summer and winter in Zhaoqing is about 22, 32 and 12 °C, respectively. Data on the prevalence of these pathogens, however, are scarce from Guangdong. Thus far, there has been only one recent study on molecular epidemiology of *Cryptosporidium* spp. in dairy cattle in the province, which identified *C. bovis*, *C. ryanae* and *C. andersoni* in pre-weaned dairy calves. There has also only been one study on *G. duodenalis* in the province, which reported a positive rate of 8.4% and identified the presence of assemblage E [47]. No studies have been conducted on *E. bieneusi* infections in pre-weaned dairy calves in this province. As *C. parvum* is still relatively uncommon in dairy cattle in China [4], and this zoonotic species has thus far not been reported in Guangdong, there is a need for more studies on the occurrence of *C. parvum*, *G. duodenalis* and *E. bieneusi* in pre-weaned dairy calves in this province.

The aim of the present study was to further examine the prevalence and identity of *Cryptosporidium* spp., *G. duodenalis* and *E. bieneusi* in pre-weaned dairy calves in Guangdong.

## Methods

### Specimen collection

A total of 388 specimens were collected from pre-weaned Holstein calves in Zhaoqing, Guangdong from March 2017 to January 2018, corresponding to spring, summer and winter seasons in the northern hemisphere. The farm that participated in this study is 0.7 km^2^ in area and had ~5000 animals at the time of the initial sampling, of which about 400 were calves. This farm adopts the intensive dairy farming system commonly seen in modern dairy operations. The cattle in the farm were grouped according to age, and all newborn calves and pre-weaned calves were kept separately from the older dairy herd. All pre-weaned calves were kept in separate stalls, which were placed adjacent to each other.

In this cross-sectional study, all pre-weaned calves on the farm were sampled on one of the three sampling dates. As there was an approximately 3-month interval between samplings, each animal participated in the study was sampled only once. Approximately 25 g fresh fecal material was collected from the rectum of each calf into 50-ml centrifuge tubes, transported to the laboratory in coolers with ice packs, and stored in 2.5% potassium dichromate at 4 °C before DNA extraction [48].

### DNA extraction

After being washed three times with distilled water by centrifugation, about 250 μl of the concentrated fecal material was used in DNA extraction with the Fast DNA SPIN Kit for Soil (MP Biomedical, Santa Ana, CA, USA), following the manufacturer-recommended procedure. DNA, eluted in 100 μl reagent-grade water, was stored at -20 °C before PCR analysis.

### PCR analysis

For the detection of *Cryptosporidium* spp., a 587-bp fragment of the small subunit (*SSU*) rRNA gene was amplified by nested PCR [49]. *Cryptosporidium* species presented were genotyped by DNA sequence analysis of positive PCR products. All *C. parvum*-positive specimens were subtyped by PCR and sequence analysis of an ~850-bp fragment of the 60-kD glycoprotein (*gp60*) gene [50]. The detection and genotyping of *G. duodenalis* were based on PCR and sequence analysis of the glutamate dehydrogenase (*gdh*), beta -giardin (*bg*) and triosephosphate isomerase (*tpi*) genes [51], whereas the prevalence and genotypes of *E. bieneusi* were identified by PCR and sequence analysis of a 392-bp fragment covering the entire internal transcribed spacer (ITS) of the rRNA gene [52]. Two replicates were used in the PCR analysis of each specimen at each locus, with reagent-grade water being used as the negative control. The secondary PCR products were examined by electrophoresis in 1.5% agarose gels and visualized after ethidium bromide staining.

### Sequence analysis

All secondary PCR products of the expected size for each genetic locus in agarose gel electrophoresis were sequenced bi-directionally with secondary PCR primers on an ABI 3730 Genetic Analyzer (Applied Biosystems, Foster City, CA, USA) at the BioSune Biotechnology Company (Shanghai, China). The sequences obtained were assembled using ChromasPro v.2.1.5.0 (http://technelysium.com.au/ChromasPro.html), edited with BioEdit v.7.1.3.0 (http://www.mbio.ncsu.edu/BioEdit/bioedit.html), and compared with reference sequences from GenBank using ClustalX 2.0.11 (http://clustal.org/) to identify the species, genotypes or subtypes of pathogens under analysis. Nucleotide sequences generated in the study were submitted to the GenBank database under the accession numbers MK252644-MK252647 for *Cryptosporidium* spp., MK252648-MK252662 and MH893662-MH893688 for *G. duodenalis*, and MK252663-MK252664 for *E. bieneusi*.

### Statistical analysis

The detection rates of each pathogen were calculated by sampling date, age and clinical sign. The Chi-square test implemented in SPSS Statistics v.20.0 for Windows (IBM Corp., New York, NY, USA) was used to compare the prevalence detection rate of pathogens among sampling dates (spring, summer and winter of northern hemisphere), age groups (weeks), or clinical signs (with or without diarrhea). Differences were considered significant at *P* < 0.05.

## Results

### Prevalence of *Cryptosporidium* spp., *G. duodenalis* and *E. bieneusi*

Of the 388 specimens analyzed, 93 (24.0%) were positive for *Cryptosporidium* spp. There were no significant differences in detection rates among the three batches of sampling conducted during the seasons (Table 1). They ranged from 12.1% (4/33) to 35.7% (5/14) in pre-weaned calves by age in weeks (Table 2).

**Table 1:** Detection rates and genotypes of *Cryptosporidium* spp., *Giardia duodenalis* and *Enterocytozoon bieneusi* in pre-weaned dairy calves in Guangdong by sampling date

Sampling date	Sample size	Detection rate of *Cryptosporidium*/*G. duodenalis*/*E. bieneusi* (%)	*Cryptosporidium* spp.			*C. parvum* subtype (*n*)	*G. duodenalis* assemblage (*n*)	*E. bieneusi* genotype (*n*)
			*C. bovis* (*n*)	*C. ryanae* (*n*)	*C. parvum* (*n*)			
First (spring)	108	31.5/64.8/38.0	28	1	5	IIdA19G1 (4)	E (65), A+E (4), A (1)	J (40), J + D (1)
Second (summer)	111	17.1/55.8/7.2	12	2	5	IIdA19G1 (5)	E (62)	J (8)
Third (winter)	169	23.1/92.3/7.1	33	4	2	IIdA19G1 (1)	E (155), A+E (1)	J (9), D (3)
Total	388	24.0/74.2/15.7	73	7	12	IIdA19G1 (10)	E (282), A+E (5), A (1)	J (57), D (3), J + D (1)

**Table 2:** Detection rates and genotypes of *Cryptosporidium* spp., *G. duodenalis* and *E. bieneusi* in pre-weaned dairy calves in Guangdong by age in weeks

Animal age (week)	Sample size	*Cryptosporidium* spp*.*				*G. duodenalis*		*E. bieneusi*	
		Detection rate (%)	*C. bovis* (*n*)	*C. parvum* (*n*)	*C. ryanae* (*n*)	Detection rate (%)	Assemblage (*n*)	Detection rate (%)	Genotype (*n*)
1	7	14.3	0	1	0	14.3	E (1)	0	–
2	14	35.7	1	4	0	28.6	E (4)	0	–
3	18	22.2	3	1	0	61.1	E (11)	11.1	J (2)
4	60	16.7	9	0	1	75.0	E (45)	10.0	J (5), D (1)
5	75	34.7	21	3	1	74.7	E (54), A + E (1), A (1)	17.3	J (13)
6	55	29.1	16	0	0	78.2	E (42), A + E (1)	9.1	J (2), D (2), J + D (1)
7	49	16.3	5	1	2	85.7	E (42)	16.3	J (8)
8	44	22.7	6	1	3	88.6	E (38), A + E (1)	20.5	J (9)
9	33	12.1	4	0	0	84.8	E (28)	24.2	J (8)
Unknown	33	27.3	8	1	0	57.6	E (17), A + E (2)	30.3	J (10)
Total	388	24.0	73	12	7	74.2	E (282), A + E (5), A (1)	15.7	J (57), D (3), J + D (1)

Based on PCR analysis at any of the three genetic loci, the detection rate of *G. duodenalis* was 74.2% (288/388). Among the three northern hemisphere seasons, the highest detection rate was 92.3% (166/169) in winter; lower rates were observed in summer (55.8% or 62/111; *χ*^2^ = 51.637, *df* = 1, *P* < 0.0001) and spring (64.8% or 70/108; *χ*^2^ = 33.155, *df* = 1, *P* < 0.0001) (Table 1). By age, detection rates increased rapidly from calves of one week in age to calves of nine weeks in age (Table 2). The detection rate was over 60% when the pre-weaned calves were three weeks old. The difference in overall detection rates between 1–2 week-old calves and older calves was significant (*χ*^2^ = 32.835, *df* = 1, *P* < 0.0001).

The overall detection rate of *E. bieneusi* was 15.7% (61/388). *Enterocytozoon bieneusi* detection appeared at older than 2-week-old calves. There was a significant difference in *E. bieneusi* detection rates between spring (38.0% or 41/108) and summer (7.2% or 8/111) (*χ*^2^ = 29.813, *df* = 1, *P* < 0.0001) or winter (7.1% or 12/169) (*χ*^2^ = 40.563, *df* = 1, *P* < 0.0001) (Table 1).

### *Cryptosporidium* spp., *G. duodenalis* and *E. bieneusi* detection and presence of diarrhea

The specimens in this study were from calves with (*n* = 21) and without diarrhea (*n* = 367). The difference in overall *Cryptosporidium* detection between the two groups of animals was not significant (*χ*^2^ = 0.817, *df* = 1, *P* = 0.24). Similarly, the difference in *G. duodenalis* (*χ*^2^ = 0.091, *df* = 1, *P* = 0.76) or *E. bieneusi* (*χ*^2^ = 0.526, *df* = 1, *P* = 0.23) detection between animals with and without diarrhea was not significant (Table 3).

**Table 3:** Detection rates and genotypes of *Cryptosporidium* spp., *Giardia duodenalis* and *Enterocytozoon bieneusi* in dairy cattle in Guangdong by diarrhea status

Animal group	Sample size	Detection rate of *Cryptosporidium*/*G. duodenalis*/*E. bieneusi* (%)	*Cryptosporidium* species			*G. duodenalis* assemblage (*n*)	*E. bieneusi* genotype (*n*)
			*C. bovis* (*n*)	*C. ryanae* (*n*)	*C. parvum* (*n*)		
Diarrhea	21	33.3/71.4/0.0	5	0	2	E (15)	–
No diarrhea	367	23.4/74.4/16.6	68	7	10	E (267), A + E (5), A (1)	J (57), D (3), J + D (1)
Total	388	24.0/74.2/15.7	73	7	12	E (282), A + E (5), A (1)	J (57), D (3), J + D (1)

### Distribution of *Cryptosporidium* species and *C. parvum* subtype

Based on sequence analysis of the *SSU* rRNA gene, three *Cryptosporidium* species were observed in pre-weaned dairy calves, including *C. bovis* (MK252645, *n* = 73), *C. parvum* (MK252646, *n* = 12) and *C. ryanae* (MK252644, *n* = 7) (Table 2). Concurrence of *C. bovis* and *C. parvum* was identified in an 8-week-old calf. *C. parvum* was the dominant species during the first two weeks of life, being detected in five of the six *Cryptosporidium*-positive calves. The appearance of *C. parvum* decreased gradually afterwards. In contrast, *C. bovis* was the dominant species at 3–9 weeks of age, reaching peak occurrence in 6-week-old calves. Similarly, *C. ryanae* was detected in 4–8-week-old calves. There was no significant association between the occurrence of diarrhea and *C. parvum* (*χ*^2^ = 0.363, *df* = 1, *P* = 0.55) or *C. bovis* (*χ*^2^ = 1.215, *df* = 1, *P* = 0.27) detection (Table 3). None of the calves with diarrhea were positive for *C. ryanae*. By sequence analysis of the *gp60* gene, 10 of the 12 *C. parvum*-positive specimens were successfully subtyped as IIdA19G1 (MK252647).

### Genotypes and subtypes of *G. duodenalis*

Of the 288 *G. duodenalis*-positive specimens, 279 were PCR positive using *bg* PCR, 209 using *tpi* and 233 using *gdh*. Two genotypes were detected among the 288 genotyped specimens, including assemblages E (*n* = 282) and A (*n* = 1); five specimens presented both assemblages (Table 1).

Sequence analysis at each genetic locus revealed a high genetic heterogeneity within assemblage E. At the *bg* locus, 15 subtypes of assemblage E were observed, including six subtypes with sequences identical to those in GenBank: E1 (MK252651, *n* = 4), E2 (MK252652, *n* = 13), E3 (MK252653, *n* = 172), E4 (MK252648, *n* = 16), E5 (MK252649, *n* = 17) and E8 (MK252650, *n* = 18). The remaining nine subtypes represented novel sequences in the literature: E18 (MH893675, *n* = 3), E19 (MH893678, *n* = 1), E20 (MH893676, *n* = 3), E21 (MH893677, *n* = 1), E22 (MH893682, *n* = 1), E23 (MH893679, *n* = 1), E24 (MH893680, *n* = 1), E25 (MH893681, *n* = 1) and E26 (MH893683, *n* = 1). In addition, double peaks were observed in the sequence trace files from 26 assemblage E specimens. Similarly, 17 assemblage E subtypes were identified at the *tpi* locus, including four known subtypes and 13 novel ones. The former known assemblages included E17 (MK252661, *n* = 118), E34 (MK252659, *n* = 14), E1 (MK252662, *n* = 12) and E18 (MK252660, *n* = 1), whereas the latter included E35 (MH893664, *n* = 3), E36 (MH893663, *n* = 3), E37 (MH893662, *n* = 20), E38 (MH893667, *n* = 1), E39 (MH893665, *n* = 1), E40 (MH893666, *n* = 1), E41 (MH893670, *n* = 1), E42 (MH893669, *n* = 4), E43 (MH893673, *n* = 2), E44 (MH893674, *n* = 1), E45 (MH893668, *n* = 2), E46 (MH893671, *n* = 1) and E47 (MH893672, *n* = 1). In contrast, 10 subtypes of assemblage E were identified at the *gdh* locus, including five known subtypes and five novel ones. The former included E1 (MK252654, *n* = 113), E3 (MK252657, *n* = 91), E29 (MK252656, *n* = 2), E37 (MK252655, *n* = 1) and E41 (MK252658, *n* = 1), whereas the latter included E35 (MH893685, *n* = 1), E36 (MH893686, *n* = 1), E38 (MH893684, *n* = 1), E39 (MH893687, *n* = 1) and E40 (MH893688, *n* = 1). Double peaks were identified from 23 and 20 assemblage E specimens at the *tpi* and *gdh* loci, respectively. The only assemblage A isolate from a 5-week-old calf was identified as subtype A1 at all three loci, representing sub-assemblage AI.

Altogether, 149 specimens were successfully subtyped at all three genetic loci, forming 49 assemblage E MLGs and one assemblage A MLG (Table 4). Because of the presence of double peaks, data from 44 specimens were excluded in the MLG data analysis. MLG-E1 and MLG-E2 were the most common MLGs in pre-weaned dairy calves, being found in 46 and 21 animals, respectively. Among sampling dates, the MLG-E1 prevalence increased from spring to winter; it was significantly different between winter and spring (*χ*^2^ = 8.6445, *df* = 1, *P* = 0.0033) and spring and summer (*χ*^2^ = 8.5132, *df* = 1, *P* = 0.0035). In contrast, there were no differences in the prevalence of MLG-E2 between spring and summer (*χ*^2^ = 0.5775, *df* = 1, *P* = 0.45), spring and winter (*χ*^2^ = 1.0172, *df* = 1, *P* = 0.31) or summer and winter (*χ*^2^ = 3.0958, *df* = 1, *P* = 0.08). There were 39 MLGs in winter but only 23 in spring and seven in summer. The number of MLGs was significantly different between spring and summer (*χ*^2^ = 10.4040, *df* = 1, *P* = 0.0012) and winter and summer (*χ*^2^ = 13.7240, *df* = 1, *P* = 0.0002).

**Table 4:** Multilocus sequence genotypes of *Giardia duodenalis* in Guangdong, China by season, based on nucleotide sequence analysis of the beta -giardin (*bg*), triosephosphate isomerase (*tpi*) and glutamate dehydrogenase (*gdh*) genes

MLGs^a^	Subtype			No. positive		
	*bg*	*tpi*	*gdh*	Spring	Summer	Winter
MLG-E1	E3	E17	E1	4	17	25
MLG-E2	E3	E17	E3	5	3	13
MLG-E3	E3	E37^b^	E1	1	1	2
MLG-E4	E3	E37^b^	E3	3	0	4
MLG-E5	E3	E34	E1	2	0	4
MLG-E6	E3	E34	E3	1	0	3
MLG-E7	E5	E17	E1	2	1	1
MLG-E8	E5	E17	E3	1	0	0
MLG-E9	E4	E17	E3	1	0	1
MLG-E10	E8	E17	E3	1	0	1
MLG-E11	E4	E17	E1	1	0	1
MLG-E12	E9	E17	E1	1	0	2
MLG-E13	E5	E36^b^	E3	1	0	0
MLG-E14	E18^b^	E17	E3	1	0	0
MLG-E15	E9	E34	E1	1	0	0
MLG-E16	E9	E17	E3	1	0	1
MLG-E17	E9	E37^b^	E1	0	0	1
MLG-E18	E9	E1	E3	0	0	2
MLG-E19	E9	E37^b^	E3	0	0	1
MLG-E20	E3	E1	E1	0	0	3
MLG-E21	E5	E36^b^	E3	1	0	0
MLG-E22	E7	E17	E3	1	0	0
MLG-E23	E3	E17	E29	0	1	0
MLG-E24	E3	E17	E39^b^	0	1	0
MLG-E25	E5	E34	E1	0	0	1
MLG-E26	E8	E35^b^	E3	1	0	0
MLG-E27	E3	E35^b^	E3	0	1	0
MLG-E28	E8	E35^b^	E1	0	0	1
MLG-E29	E8	E37^b^	E1	0	0	1
MLG-E30	E4	E37^b^	E3	0	0	1
MLG-E31	E3	E42^b^	E1	0	0	2
MLG-E32	E3	E17	E40^b^	0	0	1
MLG-E33	E3	E17	E41	0	0	1
MLG-E34	E4	E1	E1	0	0	1
MLG-E35	E3	E1	E3	0	0	1
MLG-E36	E8	E1	E1	0	0	1
MLG-E37	E3	E39^b^	E3	1	0	0
MLG-E38	E3	E45^b^	E3	0	0	1
MLG-E39	E8	E42^b^	E1	0	0	1
MLG-E40	E8	E41^b^	E3	0	0	1
MLG-E41	E3	E46^b^	E1	0	0	1
MLG-E42	E4	E43^b^	E1	0	0	1
MLG-E43	E26^b^	E17	E3	0	0	1
MLG-E44	E3	E43^b^	E3	0	0	1
MLG-E45	E7	E44^b^	E1	0	0	1
MLG-E46	E7	E1	E1	0	0	1
MLG-E47	E7	E37^b^	E1	0	0	1
MLG-E48	E3	E45^b^	E1	0	0	1
MLG-E49	E4	E17	E37	1	0	0
MLG-AI	A1	A1	A1	1	0	0

^a^MLGs were named based on sequence types at the *bg*, *tpi* and *gdh* locus. No attempts were made to consolidate MLG names of assemblage E among studies because of the extensive genetic heterogeneity within the *G. duodenalis* genotype

^b^Novel subtype

### Genotypes of *E. bieneusi*

Two known ITS genotypes were detected among the 61 successfully sequenced *E. bieneusi* specimens, including J (*n* = 57) and D (*n* = 3). Between them, J belongs to the host-adapted group 2 while D belongs to the zoonotic group 1. Concurrence of the two genotypes was seen in one animal (Table 1). Among sampling dates, genotype J was detected in all three seasons while genotype D was mostly detected in winter.

## Discussion

A high prevalence of *Cryptosporidium* spp., *G. duodenalis* and *E. bieneusi* was found in pre-weaned calves on the study farm in Guangdong. The overall detection rate of *Cryptosporidium* spp. was 24.0% (93/388). This is similar to detection rates in eastern China (19.7% or 69/350 to 24.3% or 36/148; [8, 53]) and Shaanxi (24.7% or 46/186; [9]), but higher than those reported in northwestern (10.2% or 19/186 to 15.6% or 37/237; [10, 14, 15]), southwestern (14.4% or 40/278; [13]), northern (2.6% or 9/651 to 3.47% or 14/404; [16, 17]), southern (6.4% or 19/297; [43]) and central China (15.8% or 42/265; [12]). Other studies conducted in Shanghai (37.0% or 303/818; [11]), Henan (36.2% or 108/298; [8]), and northeastern China (32.1% or 61/190 to 47.7% or 72/151; [18, 54]) reported higher detection rates of *Cryptosporidium* spp. In contrast, the 74.2% (288/388) detection rate of *G. duodenalis* obtained in this study is the highest among detection rates reported in pre-weaned dairy calves in China to date with previous studies reporting detection rates of 2.7% (11/404) to 60.1% (492/818) [12–14, 16, 17, 24, 25, 27, 29, 32, 44, 47, 55, 56]. The detection rate of *E. bieneusi* in this study was found to be 15.7% (61/388), which is within the range of reported detection rates of 2.0% (3/148) to 37.6% (35/93) in China [8, 17, 27, 36, 39–41, 46, 57]. Differences in study design, sample size, detection methods, climate, age and management of animals may be another source of variation in reported detection rates from other studies. In the present study, pre-weaned dairy calves were kept in individual stalls and under good management, with a low occurrence of diarrhea (21/388) at the sampling time. Dairy calves with diarrhea were given broad-spectrum antibiotics and oral electrolytes and were separated from healthy calves during the illness.

In the present study, seasonal differences in detection rates were observed in *G. duodenalis* and *E. bieneusi*, but not in *Cryptosporidium* spp. A higher *G. duodenalis* detection rate was noted in winter, which is consistent with findings in previous studies in Henan Province, China and in the Netherlands [32, 58]. A study of the impact of temperatures on the survival of *G. duodenalis* observed increased die off of *G. duodenalis* at higher temperatures [59], which could explain the higher transmission of *G. duodenalis* in winter in tropical Guangdong. In contrast, the detection rate of *E. bieneusi* in pre-weaned dairy calves was higher in spring than in summer and winter. This is also in agreement with observations in a previous study conducted in Korea [60]. Differences in the hardiness of environmental stages of *Cryptosporidium* spp., *G. duodenalis*, and *E. bieneusi* could contribute to the differences in seasonal transmission patterns in dairy calves among the three types of pathogens. Prevention and control measures against these enteric pathogens should take these seasonal variations in prevalence into consideration.

Three *Cryptosporidium* species were observed in pre-weaned dairy calves in Guangdong, including *C. bovis*, *C. parvum* and *C. ryanae*. *C. bovis* was the dominant species, followed by *C. parvum*. This result is different from a recent study conducted on ten farms in Guangdong, which observed only *C. bovis*, *C. ryanae* and *C. andersoni* in pre-weaned dairy calves [43]. The presence of zoonotic *C. parvum* and the absence of *C. andersoni* in pre-weaned dairy calves in this study are in stern contrast with observations in the previous study in Guangdong. However, *C. bovis* was the dominant species in both studies, which is in agreement with results in previous studies in China [4, 6]. More studies are needed to better understand the distribution of *Cryptosporidium* species in Guangdong.

The *Cryptosporidium* species distribution in pre-weaned dairy calves appears to be different between China and other countries. In a longitudinal study conducted in the USA [61], the detection rate was highest during the first four weeks, largely because of the exclusive presence of *C. parvum* which has a short patent period. In contrast, *C. bovis* appears mostly after weaning and can last for weeks or months. Because of the presence of *C. bovis*, a high overall *Cryptosporidium* detection rate was recorded throughout early life in dairy calves in a longitudinal study in Shanghai [11]. Similarly, in the present study and a previous study in Henan [7], the detection rates of *Cryptosporidium* spp. in pre-weaned dairy calves were not significantly different among age groups in weeks. The common presence of both *C. parvum* and *C. bovis* in these studies, which had different age-associated occurrence patterns in the present study, is apparently responsible for the persistent detection of *Cryptosporidium* spp. in pre-weaned dairy calves in China.

The subtype IIdA19G1 identified in the pre-weaned dairy calves in Guangdong is one of the two dominant *C. parvum* subtypes identified in ruminants, equine animals and rodents in China. To date, five *C. parvum* IId subtypes have been observed in pre-weaned dairy calves in China, including IIdA14G1, IIdA15G1, IIdA17G1, IIdA19G1 and IIdA20G1, with IIdA15G1 and IIdA19G1 being the common ones [4, 6]. In cattle, subtype IIdA19G1 has been detected in Heilongjiang, Henan, Beijing, Shanghai and Hebei and Tianjin [7, 11, 16, 17, 54] while IIdA15G1 is mostly seen in Ningxia, Sichuan, Gansu, Xinjiang, Beijing and northeastern China [10, 13, 15, 18, 62]. IId subtypes have been found in humans in Europe, Asia and Australia [63–73]. IIdA19G1, which was found in the present study, is a known human pathogen in Portugal, Sweden and China [63, 64, 71]. Both IIdA15G1 and IIdA19G1 are apparently common in various rodents in China [4].

Consistent with previous studies conducted worldwide [12, 17, 24–26, 28, 29, 74, 75], *G. duodenalis* assemblage E was the predominant assemblage identified in pre-weaned dairy calves in Guangdong. In addition, sub-assemblage AI was identified in one animal and concurrence of AI and assemblage E was identified in five animals in this study. As both assemblage E and sub-assemblage AI have been detected in humans in some studies [2, 33–35], dairy calves could play a potential role in the transmission of human *G. duodenalis* infection. Although assemblage E has not thus far been found in humans in China, more studies are needed to monitor its zoonotic transmission.

A very high genetic diversity of assemblage E was found in pre-weaned dairy calves in Guangdong. All three genetic loci in this study showed high sequence polymorphism, yielding 15, 17 and 10 subtypes at the *bg*, *tp*i and *gdh* loci, respectively. The combination of this sequence polymorphism at the three genetic loci leads to the identification of 49 MLGs of assemblage E. A previous study conducted in Shanghai also revealed high genetic diversity of assemblage E in pre-weaned dairy cattle on a single farm [24]. In that study, there were 36 MLGs of assemblage E on Farm 3. The reason for the high subtype diversity of assemblage E remains unclear. Previous studies have suggested that intra-assemblage genetic recombination may be responsible for the high subtype diversity [51, 76]. The high detection rate of *G. duodenalis* in this study and the existence of multiple subtypes on the farm and double peaks in sequencing trace files from some specimens support the potential role of genetic recombination in generating high genetic heterogeneity within assemblage E [51, 76]. This needs to be substantiated by population genetic studies.

Genotypes J and D were detected in pre-weaned dairy calves in Guangdong, with J as the dominant *E. bieneusi* genotype. This agrees with the results of most studies in China [17, 36, 39–41]. In contrast, genotype I, another common group 2 *E. bieneusi* genotype in pre-weaned dairy calves worldwide [8, 27, 37, 40–42, 77], was not detected in the present study. Genotype J has been found in a few human cases [57]. In contrast genotype D, which belongs to group 1, is a well-known zoonotic genotype [1], and appears to be a common *E. bieneusi* genotype in humans in China [63, 78, 79]. Thus far, no studies have been conducted to assess the role of zoonotic transmission of genotypes D and J in humans in Guangdong.

## Conclusions

Although *C. bovis* remains as the dominant *Cryptosporidium* species in pre-weaned dairy calves in Guangdong, there is an increasing presence of *C. parvum* in these animals, as reported elsewhere in China. The IIdA19G1 subtype of *C. parvum* identified has been detected recently in humans and other animals in China. In contrast, assemblage E of *G. duodenalis* and genotype J of *E. bieneusi* seen in this study are well-known pathogens in dairy cattle in China and elsewhere. As there are increasing numbers of reports of their detection in humans, further molecular epidemiological studies are required to better understand their transmission and public health significance in Guangdong, China.

## Abbreviations

PCR: Polymerase chain reaction
gp60: 60-kDa glycoprotein gene
MLG: Multilocus genotype
*bg*: Beta-giardia
*gdh*: Glutamate dehydrogenase
*tpi*: Triosephosphate isomerase
ITS: Internal transcribed spacer
*SSU* rRNA: Small subunit rRNA
